# Announcement: 3rd International Symposium on “Metal Elements in Environment, Medicine and Biology”

**DOI:** 10.1155/MBD.1998.171

**Published:** 1998


					3rd INTERNATIONAL SYMPOSIUM ON
"METAL ELEMENTS IN ENVIRONMENT,
MEDICINE AND BIOLOGY"
Timi,soara- Roumania, October 26-28, 1998
Organized under the auspices of:
ROUMANIAN ACADEMY- TIMISOARA BRANCH BIOCHEMICAL COMMISSION OF ROUMANIAN ACADEMY
ACADEMY OF MEDICAL SCIENCES -TIMISOARA BRANCH
Main topics
I. Researches on Metal Trace- and Macroelements in:
1. Environment
2. Human Medicine
3 Veterinary Medicine
4. Biochemistry and Molecular Biology
5. Inorganic Chemistry
6. Pharmacology
7. Nutrition and Foud Sciences
8. Biotechnology
9. Toxicology and Forensic Medicine
I1. Methods for Metal Trace- and Macroelements Determination
II1. Varia
IV. Commercial exhibitions
CORRESPONDENCE ADDRESS
Eng. FANTANA Nicu
International Symposium MEEMB '98
"Metal Elements in Environment, Medicine and Biology"
P.O. BOX 639, Postal Office 5, Piata Blcescu No. 5
RO 1900 Timi.soara, Roumania
Fax: 00 40 56 199 004
ORGANIZING COMMITTEE
Acad. Ionel HAIDUC, PhD Honorary President, Vice-president of the Roumanian Academy
Prof. Petnu DRAGAN, MD, PhD Chairman, University of Medicine and Pharmacy, Timi.soara (Roumania)
Prof. Zeno GARBAN, PhD Co-Chairman, University of Agricultural Sciences,Timi.soara (Roumania)
Prof. Eugen MAN, Eng, PhD Secretary General, University "Politehnica" Timi.soara (Roumania)
171
LOCAL COMMITTEE (Timi,soara- Roumania)
Prof. Gheorghe BACANU, MD, PhD
Assoc. Prof Gheorghe CIUHANDU, Eng.
Prof. Nicolae DOCA, Eng, PhD
.rof. ,Stefan HOLBAN, Eng. PhD
Prof. Aurel LAZUREANU, Eng, PhD
Pnof. loan MUNTEANU, MD, PhD
Senior Res.Sci. Walter SCHMIDT, Eng, PhD
Prof. loan CARTI,S, Eng, PhD
Prof Comeliu DAVIDESCU, Eng, PhD
Pnof. Constantin DOGARU, MD, PhD
Prof. Ilie JULEAN, Eng, PhD
Prof. Florin MICLEA, MD, PhD
Prof. P,un I. OTIMAN, Eng, PhD
Prof. Francisc SCHNEIDER, MD, PhD
SCIENTIFIC COMMITTEE
Prof. Manfred ANKE, PhD
Acad. Alexandru T.BALABAN, Eng, PhD
Prof. Gheorghe BENGA, MD, PhD
Assoc. Prof.Svetlana BOBCOVA, MD, PhC
Acad. Prof. Plus BRANZEU, MD, PhD
Acad. Prof. Nico! C_,AJAL, MD, PhD
Prof. Ivana DJU, PhD
Acad. Prof. Toma DORDEA, Eng, PhD
Prof. Sabin GHERGARlU, MVD, PhD
Prof. Nicolae GHILE7AN, MD, PhD
Prof. Marcel GIELEN, PhD
Prof. Wytold GRZEBISZ, PhD
Prof. Manuel J. HALPERN, MD, PhD
Prof. Sandor A. KISS, PhD
Acad. Prof. Zoran MAKSIMOVlC, PhD
Prof. Mihai NECHIFOR, MD, PhD
Prof. Istvan PALS, PhD
Prof. Sophie ERMIDOU POLLET, PhD
Senior Res.Sci.Bemd RIETZ, PhD
Prof. Peter J. SADLER, PhD
Acad. Victor E. SAHINI, PhD
Prof. Ronald SMETANA, MD, PhD
Acad. Prof. Mihai SERBAN, PhD
Prof. Andrew TAYLOR, PhD
Acad. Gheorghe ZARNEA, PhD
(Jena Germany)
(Bucure,sti Roumania)
(Cluj-Napoca Roumania)
(Chi,sin,u Moldavia)
(Timi,soara- Roumania)
(Bucure,sti Roumania)
(Beograde Jugoslavia)
(Timi,soara- Roumania)
(Cluj-Napoca Roumania)
(Cluj-Napoca- Roumania)
(Brussels- Belgium)
(Poznan Poland)
(Lisbon- Portugal)
(Szeged Hungary)
(Beograde Jugoslavia)
(la,si Romania)
(Budapest- Hungary)
(Athens- Greece)
(Roskilde- Denmark)
(Edinburgh United Kingdom)
(Bucure,sti- Roumania)
(Vienna- Austria)
(Bucure,sti- Roumania)
(Guildford- United Kingdom)
(Bucure,sti Roumania)
TEECHNICAL COMMITTEE (Timi,soara- Roumania)
Adina AVACOVICI, Researcher Gabriela DARANYI, MD, PhD
Nicu FANTANA, Eng Silviu SARAFOLEAN, Eng
Hona URZICA, student
172
PRELIMINARY PROGRAMME
Monday, October 26, 1998
10.00 20.00 Registration of participants
20.00- Welcome Reception
15.00 17.00 Sightseeing of Timi,soara
Tuesday, October 27, 1998
10.00 -10.30 Opening ceremony 10 30- 11 00- Coffee break
1 .00 -1 3.00 Plenary lectures 13.00 -14.00 Lunch
1 5.00 -1 6.00 Poster Session 16 00 17.30 Scientific Communications
18 00 Choral Concert and Organ Recital in the Roman Catholic Cathedral Timi,soara
Wednesday, October 28, 1998
9.00 -11.00 Plenary lectures
11.30 -14.00 Scientific Communications
17.00 19 00 Scientihc Communications
20.00- Farewell Party
11.00 -11.30 Coffee break
14.00 -15.00 Lunch
19.00 -18.00 Poster Session
GENERAL INFORMATION
The official language of the Symposium will be English, no simultaneous translation will be provided.
The Symposium Venue: Hotel "Timi.soara", Str. Mai Nr. 2, 1900 Timi.soara, Roumania
Registration of participants" October 26, 1998 h 10.00 20 00
October 27, 1998 h 8.00 10.00
Registration fees:
150 US$
50 US$ for participants from East European countries
150.000 lei for participants from Roumania before 15 September1998 and 180.000 lei after 15
September 1998
no fees for students
For foreign participants the payment will be made at the arrival.
Roumanian participants will make the payment by bank transfer to:
Banca Comerciala Romana, Filiala Timisoara cont 459070214801
ARRIVAL AND TRANSPORTATION
How to reach Timi.soara?
You can reach Timi.soara by plane, train or car.The main railway station is "Timi.soara Nord".
Timi.soara has an International Airport with direct flights to: Frankfurt am Main, Dusseldorf, Stuttgart
(Germany); Vienna (Austria); Verona and Rome (Italy); Chicago (USA).
It has also international connection throughout the world by the International Airport Bucharest-
Otopeni.
With "Austrian Airlines" there are direct flights from Tirni.soara to: Berlin, Dresda, Hamburg, Hannover
(Germany), Paris (France), Amsterdam (The Netherlands), Basel (Switzerland), Bologna, Firenze
(Italy), Brussels (Belgium), Kobenhavn (Denmark), Melbourne (Australia), Tokyo (Japan)
173

				

